# Associations between fine particulate matter, extreme heat events, and congenital heart defects

**DOI:** 10.1097/EE9.0000000000000071

**Published:** 2019-10-16

**Authors:** Jeanette A. Stingone, Thomas J. Luben, Scott C. Sheridan, Peter H. Langlois, Gary M. Shaw, Jennita Reefhuis, Paul A. Romitti, Marcia L. Feldkamp, Wendy N. Nembhard, Marilyn L. Browne, Shao Lin

**Affiliations:** a Department of Epidemiology, Columbia University, New York, New York; b Office of Research and Development, U.S. Environmental Protection Agency, RTP, North Carolina; c Department of Geography, Kent State University, Kent, Ohio; d Texas Department of State Health Services, Austin, Texas; e Stanford School of Medicine, Stanford, California; f National Center on Birth Defects and Developmental Disabilities, Centers for Disease Control and Prevention, Atlanta, Georgia; g Department of Epidemiology, The University of Iowa, Iowa City, Iowa; h University of Utah School of Medicine, Salt Lake City, Utah; i Departments of Pediatrics and Epidemiology, University of Arkansas for Medical Sciences, Little Rock, Arkansas; j Department of Epidemiology and Biostatistics, University at Albany, Rensselaer, New York; k New York State Department of Health, Albany, New York; l Department of Environmental Health Sciences, University at Albany, Rensselaer, New York.

## Abstract

**Background:**

Previous research reports associations between air pollution measured during pregnancy and the occurrence of congenital heart defects (CHDs) in offspring. The objective of this research was to assess if exposure to extreme heat events (EHEs) during pregnancy may modify this association.

**Methods:**

The study population consisted of 4,033 controls and 2,632 cases with dates of delivery between 1999 and 2007 who participated in the National Birth Defects Prevention Study, a multi-site case–control study in the United States. Daily data from the closest stationary fine particulate matter (PM_2.5_) monitor within 50 km from the maternal residence were averaged across weeks 3–8 post-conception. EHEs were defined as maximum ambient temperature in the upper 95th percentile for at least 2 consecutive days or the upper 90th percentile for 3 consecutive days. Logistic regression models were adjusted for maternal age, ethnicity, education, and average humidity. Relative excess risks due to interaction (RERI) were calculated.

**Results:**

Compared with women with low PM_2.5_ exposure and no exposure to an EHE, the odds of a ventricular septal defect in offspring associated with high PM_2.5_ exposure was elevated only among women who experienced an EHE (odds ratio [OR] 2.14 95% confidence interval [CI] 1.19, 3.38 vs. OR 0.97 95% CI 0.49, 1.95; RERI 0.82 95% CI −0.39, 2.17). The majority of observed associations and interactions for other heart defects were null and/or inconclusive due to lack of precision.

**Conclusions:**

This study provides limited evidence that EHEs may modify the association between prenatal exposure to PM_2.5_ and CHD occurrence.

## Abstract 2

Supplemental Digital Content is available in the text.

**What This Study Adds**

Extreme heat events are becoming more common. It is important to characterize not only their effect on health but how they may modify the effects of common coexposures, such as air pollution. We explored the joint association between fine particulate matter, extreme heat events, and congenital hearts defects in a population-based case–control study. We observed associations between ventricular septal defects and fine particulate matter were stronger when a woman also experienced an extreme heat event during early pregnancy. Findings were not consistent across other heart defects but future studies on populations with exposure to high temperatures may be warranted.

## INTRODUCTION

Congenital heart defects (CHDs) are the most common category of birth defects, with an estimated average birth prevalence of 9 per 1,000 births.^1^ While there have been major advances in the diagnosis and treatment of CHDs over the last few decades, there has been less substantial progress in identifying causes of these defects. Approximately 20% of CHDs are explained by chromosomal anomalies, syndromes, single-gene disorders or known teratogens.^2^ Researchers have examined a number of environmental and other non-genetic factors that could contribute to the large proportion of unexplained cases.^3^ Identifying modifiable risk factors for CHDs has the eventual goal of reducing CHD prevalence by informing interventions aimed at eliminating exposure to harmful agents during pregnancy. Examples of these types of potential risk factors include cigarette smoking,^4^ occupational pesticide use,^5^ and use of certain medications.^6^ Recently, studies have explored the relationship between CHDs and exposures, such as ambient air pollution and temperature, that are largely beyond an individual woman’s control but which can be addressed on the population level.

Previous research reported associations between measures of ambient air pollution during pregnancy and CHDs in offspring, although findings have been inconsistent across study populations. Researchers have observed associations between fine particulate matter (PM_2.5_) and specific types of CHDs, including coarctation of the aorta,^7^ perimembranous ventricular septal defects (VSDpm),^8^ transposition of the great arteries,^9^ and hypoplastic left heart syndrome.^10^ However, other studies did not observe those same relationships.^11–13^ There have been fewer studies that investigated the relationship between high ambient temperature and CHDs, and again results were inconsistent. In New York, Van Zutphen et al.^14^ observed null associations between extreme summer temperatures and CHDs. In Israel, Agay-Shay et al.^15^ observed a slight increase in the odds of atrial septal defects (ASDs) associated with extreme heat events (EHEs), defined in that study as temperature above the 90th percentile of the average daily temperature over the previous 90 days. Similarly, in Quebec, Auger et al.^16^ observed increased prevalence of atrial septal defects among the offspring of women who experienced elevated temperatures early in pregnancy. A recent analysis of National Birth Defects Prevention Study (NBDPS) data did not observe consistent associations between exposure to EHEs during summer and CHDs, although positive associations were observed for conotruncal heart defects, ventricular septal defects and atrial septal defects, and EHEs in the spring.^17^

To date, there have been no reports in the English-language literature of studies that attempt to simultaneously examine the impacts of both maternal exposure to ambient air pollution and higher ambient temperature on CHDs in offspring. Auger et al. and Lin et al. suggested that future research should attempt to examine the potential for air pollution to modify the relationship between temperature and CHDs.^16^ However, existing knowledge of the interplay between daily air pollution and temperature measures suggests that temperature has a direct effect on air pollution. Two examples are higher temperatures increasing ozone concentrations^18^ and temperature inversions trapping pollutants closer to the ground.^19^ Additionally, previous research has shown that temperature can modify the association between particulate matter and chronic disease morbidity, as measured by emergency department visits.^20^ Therefore, it may be more appropriate to assess how temperature could modify the association of air pollution exposure with CHDs.^21^ Expanding upon the previous NBDPS analyses conducted by Lin et al., the goal of this research was to examine the potential interaction between PM_2.5_ exposure and extreme temperatures. This would also allow us to estimate their joint association with CHDs in offspring.

## METHODS

### Study design and population

We used data from the NBDPS, a multicenter, population-based case–control study in the United States that investigates genetic and environmental risk factors for >30 major birth defects. The methods used in the NBDPS have been described previously.^22^ The NBDPS identified cases from population-based active surveillance birth defect registries in 10 US states. Cases included live births and stillbirths >20 weeks gestation or at least 500 g, as well as elective terminations of prenatally diagnosed defects when available. NBDPS study centers in several states ascertained cases statewide (AR, IA, MA), while others ascertained cases from certain counties (CA, GA, NJ, NY, NC, TX, UT). Cases were reviewed by clinical geneticists using standardized case definitions to determine study eligibility and classification. Cases with chromosomal/microdeletion disorders and disorders of known single-gene deletion causation were excluded. Controls were randomly selected in each state among live births without major defects from either hospital records or birth certificates, depending on study center. Between 6 weeks and 24 months after the estimated date of delivery (EDD), mothers were enrolled and interviewed by telephone in either English or Spanish using a structured questionnaire that covered numerous demographic, behavioral, and clinical factors before and during pregnancy.

Our analyses included CHD cases and controls from 6 participating centers (AR, CA, GA, IA, NY, TX) with EDDs from January 1, 1999, to December 31, 2007, and 2 additional states (NC and UT) that began recruitment in 2003. During this time period, the participant response was 69% among all cases and 65% for controls. As part of the original NBDPS protocol, a team of clinicians with expertise in pediatric cardiology reviewed information abstracted from medical records. They systematically assigned a single, detailed cardiac phenotype to each case whose diagnosis was confirmed by echocardiography, cardiac catheterization, surgery, or autopsy and documented in the medical record. Phenotypes were then aggregated into individual CHDs and CHD groupings.^23^ To reduce etiologic heterogeneity among CHD cases, separate analyses for the larger CHD groupings were performed. These included conotruncal heart defects, left or right ventricular outflow tract obstruction defects (LVOTO and RVOTO, respectively), and septal defects, as well as further sub-grouping for total perimembranous ventricular septal defects and atrial septal defects.

### Exposure assignment

The methods used to assign PM_2.5_ concentrations to NBDPS participants are detailed in Stingone et al.^10^ and are briefly described here. Per NBDPS protocol, the estimated date of delivery and gestational age of the offspring, from maternal interview or if not available, the medical record, was used to estimate the date of conception. That estimated date of conception was used to assign calendar dates to each week of pregnancy for NBDPS participants. Residential addresses for all centers were centrally geocoded by the Geospatial Research, Analysis, and Services Program of the U.S. Agency for Toxic Substances and Disease Registry. If a residential address could not be geocoded, a standardized protocol was followed to match the address to the closest location within a specific distance that could be geocoded. The closest intersection was attempted first, then the centroid of the ZIP Code and finally the centroid of the county. Over 80% of all NBDPS residential locations were matched to an exact USPS address. Geocoded residential addresses during weeks 3–8 of pregnancy (critical embryonic period for cardiac development) were then matched to the closest PM_2.5_ air monitor within 50 km, using monitor locations obtained from U.S. Environmental Protection Agency (EPA) Air Quality System.^24^ To be included in this analysis, women had to have a geocoded residential address with corresponding dates of residence that included weeks 3–8 of pregnancy or provide only a single address for the duration of pregnancy. Daily 24-hour PM_2.5_ measurements were averaged for weeks 3–8 of pregnancy to assign a 6-week average PM_2.5_ concentration. The 6-week average PM_2.5_ concentrations were dichotomized at the 80th percentile, based on the distribution of PM_2.5_ concentrations among the controls, to indicate high exposure. The 80th percentile was chosen as the exposure cut-point based on preliminary analyses within this study of the relationship between PM_2.5_ and odds of CHDs. The 80th percentile was equal to 17.1 µg/m^3^ of PM_2.5_, corresponding to a cut-point above the current annual U.S. EPA National Ambient Air Quality Standard.^25^

Exposure to EHEs was defined in Lin et al.^17^ and Van Zutphen et al.^14^ We collected meteorological data, including daily temperature, dew point, wind speed, and atmospheric pressure for each participating NBDPS study center from the National Center for Atmospheric Research. Geocoded maternal residences were linked with the closest weather station and assigned meteorological data from that station. Duration of EHEs was defined in 2 ways: (1) at least 2 consecutive days with the daily maximum temperature (Tmax) above the 95th percentile of the Tmax distribution for the season and year (EHE95); (2) at least 3 consecutive days with daily Tmax above the 90th percentile of the Tmax distribution for the season and year (EHE90).

### Statistical analysis

For consistency between studies, the same set of confounding variables, variable constructions and study exclusions defined in Lin et al^17^ was used in this analysis. Briefly, Lin et al^17^ evaluated the following variables obtained from the maternal interview as potential confounders for the relationship between EHE and CHDs: maternal race/ethnicity (nonHispanic [NH] white, NH-black, Hispanic, other); maternal education level at delivery (<12, ≥12 years), maternal age at delivery (≤19, 20 – 34, ≥35 years), parity (0, 1, ≥2); prenatal care (yes/no); folic acid intake (yes/no); pre-pregnancy body mass index (underweight, normal weight, overweight, obese); maternal medical conditions such as fever, hypertension, pregestational diabetes and gestational diabetes (yes/no); family history of CHDs (yes/no); use of hot bath/tub during pregnancy (yes/no); diuretic/laxative medication during pregnancy (yes/no); dietary caffeine consumption (>100, ≤100 mg/day); alcohol consumption (yes/no); and smoking (yes/no). Additionally, dew point, a better indicator of moisture in the air than relative humidity, was evaluated as a potential confounder. No variable led to a 10% change of the point estimate (all <2%) in models examining EHEs. Subsequently, a directed acyclic graph was constructed using the potential confounders described above, and maternal age, maternal education, race/ethnicity, and dew point were identified as confounders. Those 4 variables were then used as the adjustment set for all models.

Multivariable logistic regression models were constructed to obtain adjusted odds ratios (ORs) and 95% confidence intervals (CIs) for the joint association of PM_2.5_ and exposure to EHEs. Two sets of models were used to assess interaction on the multiplicative scale and the additive scale. Separate models were also created for the 2 different metrics for estimating exposure to EHEs. Models were constructed for the full population and then for 3 subpopulations defined by the season of exposure in early pregnancy: (1) women who had at least 1 day of the critical period of CHD embryogenesis (postconceptional weeks 3–8) in the summer or spring seasons; (2) women who had their entire critical period within the spring or summer season; and (3) women who had at least 1 day of the critical period in the summer season. Season was defined consistently for each NBDPS study center as the months of June, July, and August for summer, and March, April, and May for spring. The spring and summer seasons were examined because the highest average temperatures across all sites occur during these seasons. Models were adjusted for the confounders identified above, and the full population model was also adjusted for an indicator of having at least 1 day of pregnancy within the spring or summer seasons.

Interactions on the multiplicative scale were assessed by including an interaction term between the indicator variables for high PM_2.5_ exposure and exposure to an EHE within the logistic regression model. Likelihood ratio tests, using an alpha level of 0.1, were conducted to determine if there was evidence of effect measure modification. Given the number of cases and controls within our analysis, we expect that using a greater Type I error rate (α = 0.1) to detect interactions will result in a useful gain in power.^26^ Not relying solely on statistical significance, we also examined the stratum-specific estimates to assess the presence of interaction on the multiplicative scale. Within each stratum of presence of an EHE, the reference group was the women with low PM_2.5_ exposure.

Interactions on the additive scale between PM_2.5_ and EHEs were assessed by constructing a 4-level variable that represented the joint exposure of PM_2.5_ and EHEs and then calculating the relative excess risk due to interaction (RERI). The equation for calculating the RERI is as follows: RERI = OR_11_ − OR_10_ − OR_01_ + 1, where the 3 ORs are obtained using the 4-level variable representing the joint exposure of PM_2.5_ and EHEs. The reference group used to obtain the ORs consisted of the participants with PM_2.5_ exposure lower than the 80th percentile of the PM_2.5_ distribution among all of the controls and no EHE during the critical window of pregnancy. OR_11_ is the estimate for women with the highest levels of PM_2.5_ and an EHE during the critical window of pregnancy. OR_10_ is the estimate for women with the highest levels of PM_2.5_ and no EHE during pregnancy, while OR_01_ is the estimate for women with lower levels of PM_2.5_ and an EHE during pregnancy. Corresponding likelihood-based 95% CIs for the RERI were calculated.^27^

To focus on women with the greatest probability of experiencing high temperatures, analyses were repeated in the subpopulation of women who lived in the South (AR, TX) and Southeast (GA, NC) climate regions^28^ and whose critical period was entirely within the spring and summer season. Analyses were performed using SAS version 9.4. The NBDPS was approved by the Institutional Review Boards of all participating centers and all participants provided consent prior to participation. Replication of selected analyses were performed and results compared in order to ensure data quality.

## RESULTS

Our analyses included 4,033 controls and 2,632 CHD cases for which at least 1 geocoded residence was available and that residence was within 50 km of at least 1 PM_2.5_ monitor. The number of cases for each CHD grouping ranged from 447 (RVOTO) to 958 (septal defects) (Table 1). Sample size allowed for the analysis of 2 individual septal defect phenotypes: ASD (n = 420) and VSDpm (n = 407). The demographic characteristics of the final analytic population are presented in Table 1.

**Table 1:**
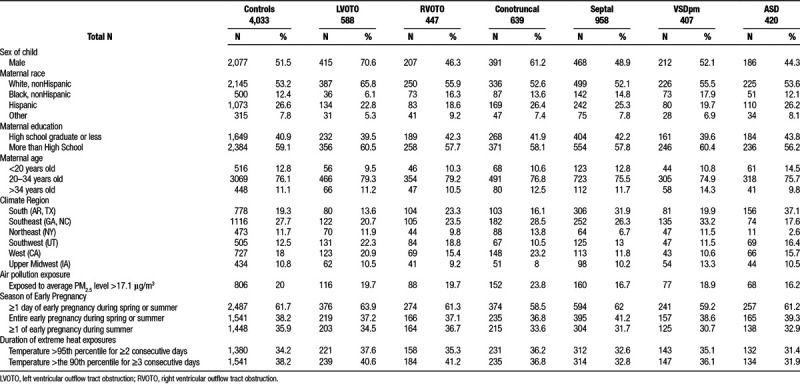
Demographic and exposure profiles of cases and controls, NBDPS 1999–2007

A majority of the population was was NH-white, had more than a high school education and was between the ages of 20 and 34. About 60% of mothers had at least 1 day of early pregnancy during spring or summer, and >30% had at least one day of early pregnancy in summer. Between 16% and 24% of mothers were assigned PM_2.5_ concentrations >17.1 µg/m^3^, the cut-point used to define high PM_2.5_ exposure in our analyses. Thirty to forty percent of mothers experienced an EHE during early pregnancy.

Estimates of the association between PM_2.5_ and CHDs, stratified by the presence of an EHE defined using the 2-day definition, are presented in Figure. Complete numeric results are available in the eTable; http://links.lww.com/EE/A63. These results were produced by the multiplicative models. We observed similar results for the combined effect of PM_2.5_ exposure and EHE95s on CHDs when we included the full population in the analysis, and when we limited the analysis to the sub-group that had part or the entire critical window for cardiogenesis in the warmer seasons. Results were not consistent across the individual defects. For example, the odds of delivering a child with a VSDpm were slightly, but consistently higher among women with greater PM_2.5_ exposure who were also exposed to an EHE. Statistical interaction was observed in both the full population and in the subpopulation with at least 1 day in either the spring or summer season. The effect estimate with the largest magnitude was observed among women with at least 1 day of the critical window within the summer season. Among women exposed to an EHE, women exposed to high levels of PM_2.5_ had 1.59 (95% CI 0.94, 2.71) times the odds of having a child with a VSDpm than women with low exposure to PM_2.5_, while among women unexposed to an EHE, the association between PM_2.5_ and VSDpm was estimated at 0.97 (95% CI 0.49, 1.95). A different pattern was observed among RVOTO defects, with women exposed to EHE95s having reduced odds of defects associated with exposure to PM_2.5_ (OR 0.56; 95% CI 0.31, 1.00). We also observed reduced odds of septal defects, particularly ASDs, associated with high PM_2.5_ exposure among women not exposed to EHEs across the different subpopulations.

**Figure.**
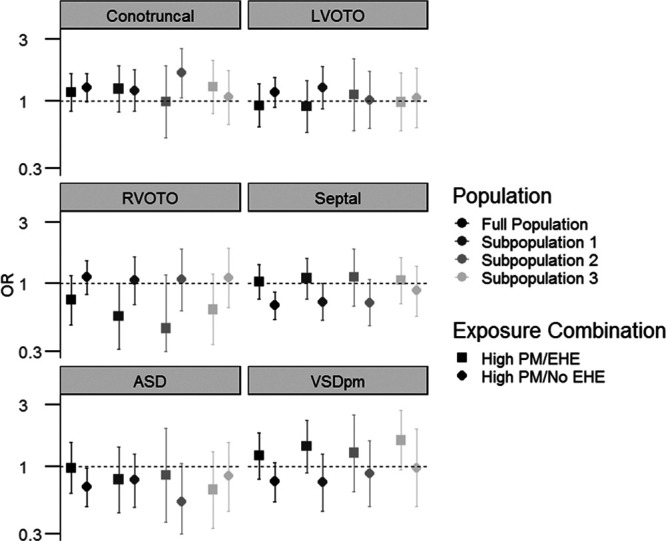
Adjusted ORs and 95% CI for the effect of high PM_2.5_ exposure on CHDs with exposure to EHEs and without exposure to EHEs. EHE defined as temperature greater than the 95th percentile for at least 2 consecutive days; Subpopulation 1 = at least 1 day of early pregnancy in spring or summer season; Subpopulation 2 = entire early pregnancy in spring or summer season. Subpopulation 3 = at least 1 day of early pregnancy in summer season.; Complete quantitative results can be found in eTable; http://links.lww.com/EE/A63. LVOTO, left ventricular outflow tract obstruction; RVOTO, right ventricular outflow tract obstruction.

A notable exception to the similar patterns observed across subpopulations occurred for conotruncal defects (Figure, eTable; http://links.lww.com/EE/A63). Associations between PM_2.5_ and conotruncal defects looked similar, regardless of the presence of an EHE95 in all subpopulations except the subpopulation of women whose entire critical window was within the spring or summer seasons. Women in this subpopulation had elevated odds of having a child with a conotruncal defect only when an EHE did not occur (1.64 [95% CI 1.06, 2.53] vs. 0.99 [95% CI 0.52, 1.88]), although there was no formal evidence of statistical interaction (*P* = 0.2). Results across CHDs and subpopulations were generally consistent when using EHE90 (eTable; http://links.lww.com/EE/A63).

Similar patterns were observed when examining results from additive models with the 4-level joint variable of PM_2.5_ exposure and EHE95s (Table 2). We observed elevated odds for VSDpm (OR: 2.14; 95% CI: 1.19, 3.83) when mothers with at least 1 day of early pregnancy in the summer season experienced both high PM_2.5_ exposure and an EHE95 during early pregnancy. Although interaction was not statistically significant, the opposite was observed when examining RVOTO defects. This was particularly observed when looking at women whose entire critical window occurred within the spring or summer seasons (OR 0.50; 95% CI 0.20, 1.28). The magnitudes of RERIs were consistently positive across subpopulations for VSDpm, and consistently negative for RVOTO defects (Table 2 and eFigure; http://links.lww.com/EE/A64). Elevated associations for VSDpm did not persist with EHE90, with the heat event defined as temperature greater than the 90th percentile for at least 3 consecutive days (eTable; http://links.lww.com/EE/A65). We did not observe evidence of interaction for other CHDs.

**Table 2:**
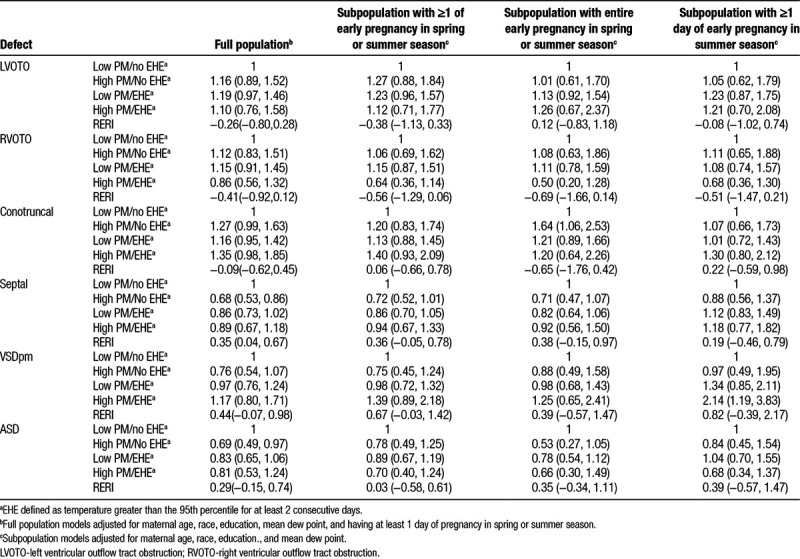
Adjusted ORs, 95% CI and relative excess risks due to interaction for the joint association between PM_2.5_ exposure, EHEs, defined as temperature above the 95th percentile for ≥2 days, and CHDs (additive interactions) in the NBDPS, 1999–2007

Additional analyses examining women in the climate regions most likely to be exposed to high absolute temperatures (i.e., AR, NC, GA, TX) who had their entire critical window within the spring or summer seasons provided imprecise estimates due to the reduced sample size (Table 3). Although we observed increased odds of LVOTO defects in the South climate region among women with high PM_2.5_ exposure and exposure to an EHE95, the imprecision of the estimates (as evidenced by the wide confidence intervals) reinforces the need for cautious interpretation of these results. We continued to see patterns of greater odds of septal defects, particularly ASDs, and reduced odds of RVOTO defects among women exposed to PM_2.5_ and EHE95s in the Southeast region. In the South region, we observed consistently greater odds of conotruncal defects for all exposure categories, when comparing with the referent of women with low PM_2.5_ and no EHEs.

**Table 3:**
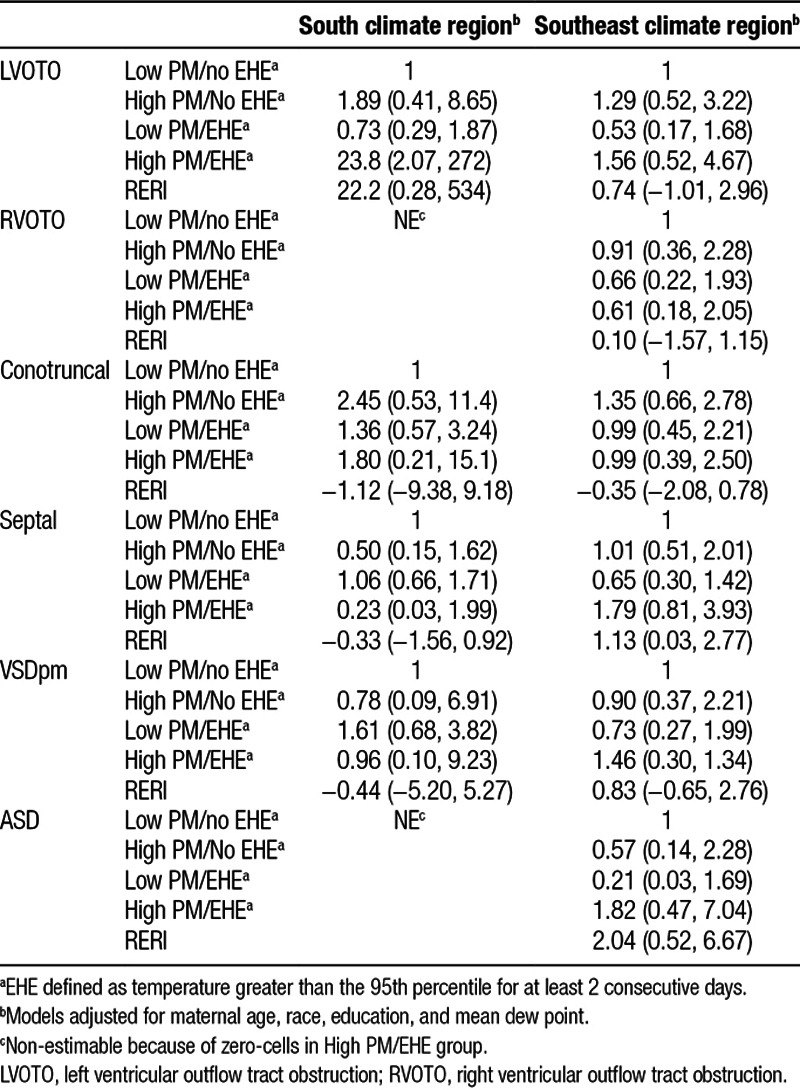
Adjusted ORs, 95% CI, and RERI for the relationship between PM_2.5_, EHE, and select CHDs among women whose first 8 weeks of pregnancy occurred in the spring/summer season within the South and Southeast Climate Regions

## DISCUSSION

Building on previous analyses that have reported observations between PM_2.5_ exposure and CHDs, we evaluated how exposure to EHEs during early pregnancy might modify these associations. We observed significant evidence for interaction on the multiplicative scale between PM_2.5_ exposure and the presence of EHEs on the odds of VSDpm defects and RVOTO defects, particularly when at least 1 day of the critical period for cardiogenesis occurred during the warmer seasons of spring and summer. While the odds of having VSDpm defects were higher in the presence of both an EHE and higher PM_2.5_ exposure, we observed odds of RVOTO defects that were lower in the presence of both an EHE and higher PM_2.5_ exposure. Similarly, when constructing models to assess departures from additive interaction, we observed positive RERIs and elevated odds ratios for VSDpm defects when women had greater PM_2.5_ exposure and experienced an EHE. Again, this was more pronounced when the women had at least part of the critical window within the warmer seasons. We also observed negative RERIs and reduced odds of RVOTO defects among women with higher PM_2.5_ and an EHE, when compared with women with low PM_2.5_ and no EHE. The largest positive RERI was observed when examining LVOTO defects within the population that lives in the South climate region, although these results are imprecise due to small sample size. Given the number of comparisons made in this study, we recommend caution when interpreting the results of the few positive associations observed.

Two recent studies have evaluated the effect of EHEs on birth defects within our study population of the NBDPS. Soim et al.^29^ did not observe associations between EHEs during early pregnancy and neural tube defects. Lin et al. observed elevated odds ratios for VSDpm defects in the full population and for VSDpm and conotruncal defects in the South climate region.^17^ We are unaware of any previous research that examines the interaction of ambient air pollution and temperature on birth defect phenotypes, and only a few studies have examined the interaction of ambient air pollutants and temperature on other birth outcomes. Schifano et al.^30,31^ observed increased risks of preterm birth during heat waves and with maximum apparent temperature, as well as with increases in PM_10_ and NO_2_ concentrations. However, they did not evaluate the data for interactions or potential effect measure modification between air pollution and temperature. Their results suggest it is plausible that air pollution and temperature could interact to contribute to adverse effects during pregnancy.

Toxicological evidence suggests that exposure to high ambient temperatures can intensify the effects of environmental chemicals.^32^ A number of physiologic changes that occur during higher temperatures, such as increased ventilation, can play a role in increasing the biological dose of an air pollutant that is experienced among exposed populations. Additionally, evidence from animal studies suggest that, while increasing ambient temperature increases the rate of detoxification, it also increases the toxicity of the agent.^33^ The results from this epidemiologic study are preliminary, and we are not able to draw causal conclusions regarding the presence of biological interaction between air pollution and EHEs. However, the biological plausibility of the interaction and the increasing prevalence of EHEs suggest that additional, more targeted studies of the role that air pollution and temperature play in adverse birth outcomes such as CHDs are warranted.

The NBDPS provided an advantageous study population and design to assess the relationship between PM_2.5_, EHEs, and CHDs. This population-based case–control study systematically classified CHDs, which reduced the possibility of outcome misclassification. It also collected detailed residential history during pregnancy that allowed linkage with temporally relevant exposure data. The relatively large sample size of CHDs facilitated the investigation of relationships between PM_2.5_, temperature and CHDs within subpopulations of women at greater likelihood of being exposed to high temperatures. It was also possible to restrict the population further and conduct preliminary analyses on specific climate regions that have warmer absolute temperatures. However, small sample sizes after these multiple stratifications hindered drawing any strong conclusions. The inclusion of participants from multiple sites across the United States provided clear gradients in PM_2.5_ exposure and occurrence of EHEs. However, we did not have adequate sample size within each site to obtain site-specific estimates. We did not assess other traffic-related pollutants or neighborhood measures of socioeconomic status. Both of these spatially-varying factors may also be associated with CHDs, and thus may contribute to the associations observed within this study.

It is important to consider that the reliance on ambient measures of air pollution could lead to differential misclassification based on temperature status. For example, during an EHE, women may be more likely to stay indoors in air conditioning. Thus, an ambient measure of PM_2.5_ may be less representative of a woman’s actual exposure than it would be when an EHE does not occur. We did not have information on time spent outdoors, and this misclassification could lead to bias in our results. Additionally, we used the closest monitor within 50 km to assign exposure to PM_2.5_. Using the closest monitor could have led to misclassification of exposure, as absolute distance does not necessarily reflect dispersion of pollutants in a given area. Although the median distance from a woman’s residence to a PM_2.5_ monitor was ~10 km, the 50 km buffer size may also have led to some misclassification of exposure, as those living far from the monitor may not be well-represented by the pollutant concentrations at the location of the monitor. Similar misclassification could have occurred for temperature. The average distance to a weather station varied slightly by region, with women in the Northeast region living closer to a weather station and women in the Southeast living further away. Previous sensitivity analyses stratified the population by distance to the closest weather station and observed only slight differences in the estimated effects of extreme heat events.^17^ There is also the potential for temporal misclassification as the calendar dates of gestation used to assign exposure were based on estimated dates of conception. This misclassification is likely non-differential with respect to both exposure and outcome status.

As one of the first studies to assess the interaction between air pollution and temperature on birth defects, we conducted a broad analysis of several CHD groupings. These lead to a large number of analytic results. Because of the potential for exposure misclassification and the number of effect estimates calculated in the study, it is not possible to rule out the role of random variation in our results. Future studies that are able to follow-up on our results with more targeted analyses of these identified associations, potentially using improved exposure metrics and larger sample sizes to ensure appropriate power to obtain more precise estimates, would be informative. Using larger sample sizes would also allow for a more detailed investigation of the interplay between temperature, humidity, and air pollution as contributors to adverse outcomes, as opposed to our approach of adjusting models for the effects of humidity.

This study provides limited evidence of interaction between PM_2.5_ exposure and the presence of extreme heat events during pregnancy and CHDs in offspring. There is some suggestion that observed relationships between PM_2.5_, EHEs, and CHDs may be stronger among women whose early pregnancy occurs in the spring and summer months and in the South and Southeast climate regions of the United States. Future studies that focus on these potentially higher-risk populations may be warranted.

## Conflict of interest statement

The authors declare that they have no financial conflict of interest with regard to the content of this report.

## Acknowledgements

We thank Eugene Wong for conducting an analytic replication for the manuscript. The authors also thank the study participants.

Availability of Data and Code: Access to data from the National Birth Defects Prevention Study can be obtained by completing a collaboration request form, available at https://www.cdc.gov/ncbddd/birthdefects/nbdps-public-access-procedures.html.

## Supplementary Material
