# Supplementary material for: Associations between fine particulate matter, extreme heat events, and congenital heart defects
Source: Environ Epidemiol. 2019 Oct 16;3(6):e071. doi: 10.1097/EE9.0000000000000071 (PMC7004451; doi:10.1097/EE9.0000000000000071)
Supplement: Supplementary file 1 [file ee9-3-e071-s001.pdf]

Table: Adjusted odds ratios and 95% confidence intervals looking at the effect of high PM exposure on congenital heart defects within strata of exposure to heat waves, NBDPS 1999-2007.

Defect		Full Population[a]	p-value	Subpopulation with at least one day of early pregnancy in spring or summer season[b]	p-value	Subpopulation with entire early pregnancy in spring and summer season[b]	p-value	Subpopulation with at least one day of early pregnancy in summer season[b]	p-value
LVOTO	Effect of high PM among those exposed to heat wave (definition 1)	0.92 (0.63, 1.35)	0.33	0.91 (0.57, 1.44)	0.27	1.12 (0.59, 2.13)	0.81	0.98 (0.59, 1.64)	0.84
	Effect of high PM among those not-exposed to heat wave (definition 1)	1.16 (0.89, 1.52)		1.27 (0.87, 1.84)		1.01 (0.61, 1.70)		1.06 (0.62, 1.79)	
RVOTO	Effect of high PM among those exposed to heat wave (definition 1)	0.75 (0.48, 1.16)	0.13	0.56 (0.31, 1.00)	0.08	0.45 (0.17, 1.17)	0.12	0.63 (0.34, 1.18)	0.17
	Effect of high PM among those not-exposed to heat wave (definition 1)	1.12 (0.83, 1.51)		1.06 (0.69, 1.62)		1.08 (0.62, 1.86)		1.11 (0.65, 1.88)	
Conotruncal	Effect of high PM among those exposed to heat wave (definition 1)	1.16 (0.83, 1.62)	0.67	1.24 (0.82, 1.86)	0.92	0.99 (0.52, 1.88)	0.2	1.28 (0.80, 2.05)	0.58
	Effect of high PM among those not-exposed to heat wave (definition 1)	1.27 (0.99, 1.63)		1.20 (0.83, 1.74)		1.64 (1.06, 2.53)		1.07 (0.66, 1.73)	
Septal	Effect of high PM among those exposed to heat wave (definition 1)	1.03 (0.76, 1.40)	0.03	1.10 (0.76, 1.57)	0.09	1.12 (0.67, 1.86)	0.17	1.06 (0.70, 1.60)	0.53
	Effect of high PM among those not-exposed to heat wave (definition 1)	0.68 (0.53, 0.86)		0.72 (0.52, 1.01)		0.71 (0.47, 1.07)		0.88 (0.56, 1.37)	

VSDpm	Effect of high PM among those exposed to heat wave (definition 1)	1.21 (0.80, 1.81)	0.08	1.43 (0.89, 2.28)	0.06	1.27 (0.64, 2.51)	0.42	1.59 (0.94, 2.71)	0.26
	Effect of high PM among those not-exposed to heat wave (definition 1)	0.76 (0.53, 1.07)		0.75 (0.45, 1.24)		0.88 (0.49, 1.58)		0.97 (0.49, 1.95)	
ASD	Effect of high PM among those exposed to heat wave (definition 1)	0.97 (0.62, 1.53)	0.23	0.79 (0.44, 1.41)	0.97	0.85 (0.37, 1.96)	0.39	0.66 (0.33, 1.29)	0.6
	Effect of high PM among those not-exposed to heat wave (definition 1)	0.69 (0.49, 0.97)		0.78 (0.49, 1.25)		0.53 (0.27, 1.05)		0.84 (0.45, 1.54)	
LVOTO	Effect of high PM among those exposed to heat wave (definition 2)	0.99 (0.70, 1.41)	0.59	1.00 (0.65, 1.52)	0.51	1.05 (0.54, 2.02)	0.98	0.98 (0.59, 1.62)	0.85
	Effect of high PM among those not-exposed to heat wave (definition 2)	1.12 (0.84, 1.49)		1.21 (0.81, 1.80)		1.06 (0.63, 1.76)		1.05 (0.60, 1.82)	
RVOTO	Effect of high PM among those exposed to heat wave (definition 2)	0.71 (0.47, 1.07)	0.05	0.57 (0.33, 0.98)	0.06	0.45 (0.18, 1.17)	0.11	0.67 (0.36, 1.22)	0.25
	Effect of high PM among those not-exposed to heat wave (definition 2)	1.20 (0.88, 1.64)		1.11 (0.71, 1.73)		1.09 (0.63, 1.90)		1.08 (0.62, 1.87)	
Conotruncal	Effect of high PM among those exposed to heat wave (definition 2)	1.02 (0.73, 1.43)	0.16	1.05 (0.69, 1.60)	0.35	0.71 (0.33, 1.54)	0.05	1.13 (0.70, 1.83)	0.98
	Effect of high PM among those not-exposed to heat wave (definition 2)	1.38 (1.08, 1.77)		1.37 (0.95, 1.97)		1.71 (1.12, 2.59)		1.12 (0.70, 1.840)	
Septal	Effect of high PM among those exposed to heat wave (definition 2)	0.94 (0.70, 1.28)	0.17	1.03 (0.72, 1.49)	0.24	0.89 (0.49, 1.61)	0.72	1.08 (0.71, 1.65)	0.28

	Effect of high PM among those not-exposed to heat wave (definition 2)	0.72 (0.57, 0.92)		0.77 (0.55, 1.07)		0.78 (0.53, 1.15)		0.78 (0.51, 1.20)	
VSDpm	Effect of high PM among those exposed to heat wave (definition 2)	1.10 (0.73, 1.64)	0.25	1.34 (0.83, 2.17)	0.17	1.02 (0.46, 2.24)	0.95	1.59 (0.92, 2.77)	0.18
	Effect of high PM among those not-exposed to heat wave (definition 2)	0.81 (0.57, 1.14)		0.84 (0.52, 1.35)		0.99 (0.58, 1.69)		0.94 (0.49, 1.78)	
ASD	Effect of high PM among those exposed to heat wave (definition 2)	0.80 (0.51, 1.27)	0.9	0.76 (0.43, 1.34)	0.85	0.58 (0.20, 1.66)	0.91	0.69 (0.35, 1.36)	0.88
	Effect of high PM among those not-exposed to heat wave (definition 2)	0.77 (0.55, 1.08)		0.81 (0.50, 1.30)		0.62 (0.34, 1.14)		0.74 (0.40, 1.38)	

Abbreviations: ASD-atrial septal defect; LVOTO-left ventricular outflow tract obstruction; RVOTO-right ventricular outflow tract obstruction; VSDpm-perimembranous ventricular septal defect

[a]Full population models adjusted for maternal age, race, education and having at least one day of pregnancy in spring or summer season

[b]Subpopulation models adjusted for maternal age, race and education

definition 1: temperature greater than the 95th percentile for at least 2 consecutive days

definition 2: temperature greater than the 90th percentile for at least 3 consecutive days
