# Supplementary material for: Associations between fine particulate matter, extreme heat events, and congenital heart defects
Source: Environ Epidemiol. 2019 Oct 16;3(6):e071. doi: 10.1097/EE9.0000000000000071 (PMC7004451; doi:10.1097/EE9.0000000000000071)
Supplement: Supplementary file 2 [file ee9-3-e071-s002.pdf]

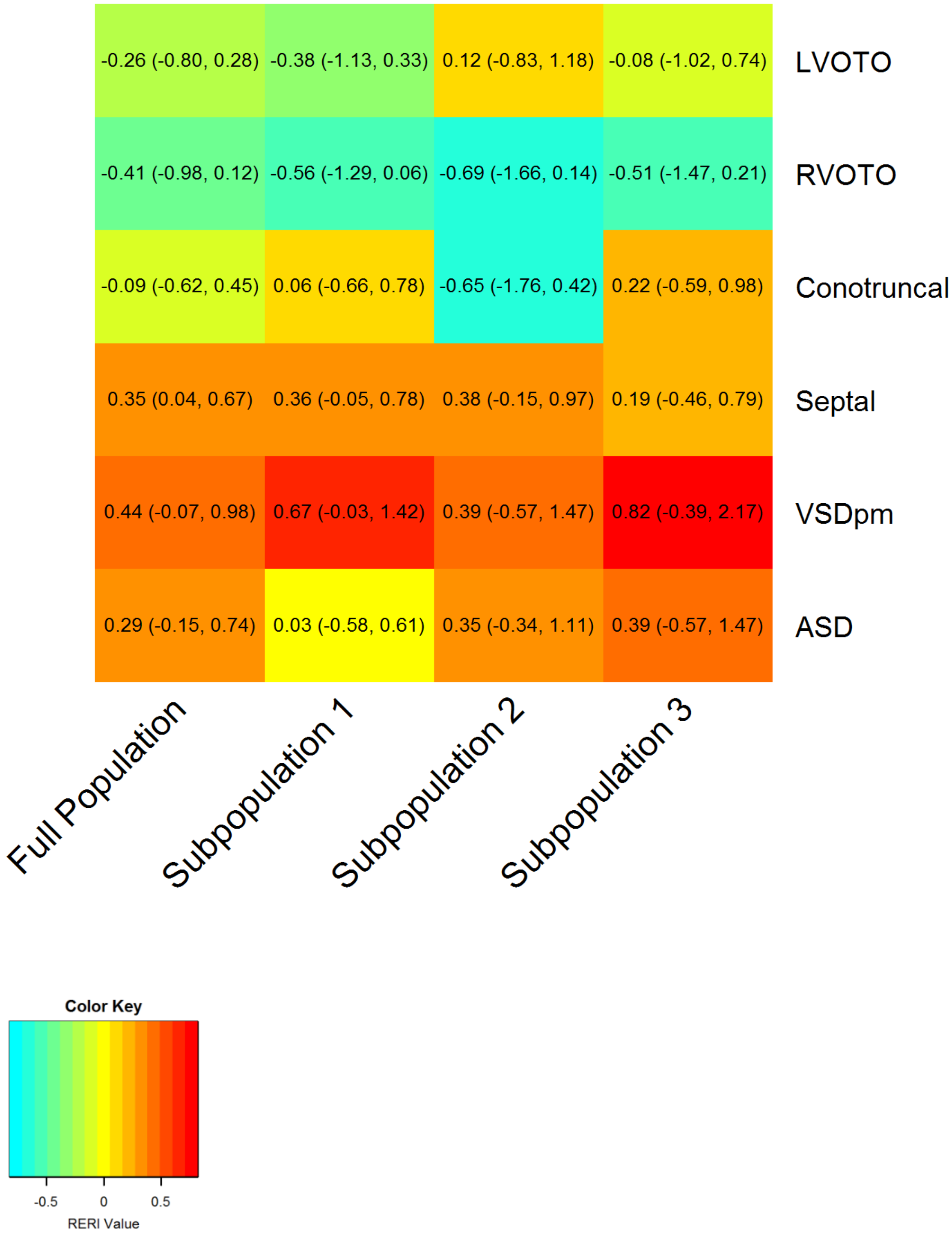

Figure: Relative excess risks due to interaction and 95% confidence intervals between $PM_{2.5}$, extreme heat events and congenital heart defects, National Birth Defects Prevention Study 1999-2007. Subpopulation 1= at least one day of early pregnancy in spring or summer season; Subpopulation 2= entire early pregnancy in spring or summer season. Subpopulation 3= at least one day of early pregnancy in summer season. Abbreviations: ASD: Atrial septal defect, LVOTO: left ventricular outflow tract obstruction, RERI: relative excess risk due to interaction, RVOTO: right ventricular outflow tract obstruction, VSDpm: perimembranous ventricular septal defect.
