# Supplementary material for: Associations between fine particulate matter, extreme heat events, and congenital heart defects
Source: Environ Epidemiol. 2019 Oct 16;3(6):e071. doi: 10.1097/EE9.0000000000000071 (PMC7004451; doi:10.1097/EE9.0000000000000071)
Supplement: Supplementary file 3 [file ee9-3-e071-s003.pdf]

Table: Adjusted odds ratios, 95% confidence intervals and relative excess risks due to interaction for joint association between PM exposure, extreme heat events, defined as above the 90th centile of temperature for 3 consecutive days, and birth defects, NBDPS 1999-2007.

Defect		Full Population[a]	Subpopulation with at least one day of early pregnancy in spring or summer season[b]	Subpopulation with entire early pregnancy in spring or summer season[b]	Subpopulation with at least one day of early pregnancy in summer season[b]
LVOTO	Low PM/no EHE	1	1	1	1
	High PM/No EHE	1.12 (0.84, 1.49)	1.21 (0.82, 1.80)	1.06 (0.63, 1.76)	1.05 (0.61, 1.82)
	Low PM/EHE	1.12 (0.91, 1.37)	1.13 (0.88, 1.43)	1.08 (0.79, 1.47)	1.16 (0.80, 1.67)
	High PM/EHE	1.11 (0.78, 1.55)	1.12 (0.73, 1.72)	1.13 (0.59, 2.17)	1.13 (0.65, 1.96)
	RERI	-0.15 (-0.67, 0.35)	-0.21 (-0.94,0.45)	-0.01 (-0.94, 0.99)	-0.07 (-1.02,0.70)
RVOTO	Low PM/no EHE	1	1	1	1
	High PM/No EHE	1.20 (0.88, 1.64)	1.11 (0.71, 1.74)	1.10 (0.63, 1.90)	1.08 (0.62, 1.87)
	Low PM/EHE	1.28 (1.02, 1.61)	1.20 (0.92, 1.59)	1.17 (0.83, 1.66)	1.01 (0.68, 1.50)
	High PM/EHE	0.91 (0.61, 1.36)	0.68 (0.40, 1.19)	0.53 (0.21, 1.36)	0.68 (0.36, 1.28)
	RERI	-0.56 (-1.17, -0.02)	-0.63 (-1.41,0.01)	-0.73 (-1.75,0.12)	-0.41 (-1.38,0.28)
Conotruncal	Low PM/no EHE	1	1	1	
	High PM/No EHE	1.38 (1.08, 1.77)	1.37 (0.95, 1.97)	1.71(1.12, 2.59)	1.12 (0.70, 1.81)
	Low PM/EHE	1.04 (0.85, 1.28)	0.98 (0.77, 1.25)	0.97 (0.71, 1.32)	0.82 (0.58, 1.16)
	High PM/EHE	1.07 (0.78, 1.47)	1.03 (0.68, 1.56)	0.69 (0.32, 1.47)	0.93 (0.56, 1.54)
	RERI	-0.35(-0.86,0.13)	-0.32(-1.03,0.31)	-0.99 (-2.02,-0.10)	-0.02 (-0.79,0.61)
Septal	Low PM/no EHE	1	1	1	
	High PM/No EHE	0.72 (0.57, 0.92)	0.77 (0.55, 1.07)	0.78 (0.53, 1.15)	0.78 (0.51, 1.20)
	Low PM/EHE	0.74 (0.63, 0.88)	0.67 (0.55, 0.82)	0.62 (0.48, 0.80)	0.70 (0.53, 0.94)
	High PM/EHE	0.70 (0.53, 0.93)	0.69 (0.49, 0.99)	0.55 (0.31, 0.98)	0.76 (0.49, 1.18)
	RERI	0.23 (-0.05, 0.51)	0.25 (-0.12, 0.60)	0.15 (-0.32, 0.61)	0.28 (-0.22, 0.71)
VSDpm	Low PM/no EHE	1	1	1	
	High PM/No EHE	0.81 (0.57, 1.14)	0.84 (0.52, 1.35)	0.99 (0.58, 1.69)	0.94 (0.49, 1.78)
	Low PM/EHE	0.88 (0.69, 1.12)	0.75 (0.56, 1.02)	0.78 (0.53, 1.13)	0.84 (0.53, 1.31)
	High PM/EHE	0.96 (0.66, 1.40)	1.01 (0.63, 1.60)	0.79 (0.37, 1.70)	1.33 (0.74, 2.41)
	RERI	0.28 (-0.19, 0.76)	0.42 (-0.21, 1.03)	0.03 (-0.84, 0.90)	0.56 (-0.38, 1.46)
ASD	Low PM/no EHE	1	1	1	
	High PM/No EHE	0.77 (0.55, 1.08)	0.81 (0.50, 1.30)	0.62 (0.34, 1.14)	0.74 (0.40, 1.38)
	Low PM/EHE	0.75 (0.59, 0.96)	0.77 (0.58, 1.02)	0.61 (0.42, 0.87)	0.78 (0.52, 1.17)
	High PM/EHE	0.60 (0.39, 0.93)	0.58 (0.33, 1.01)	0.35 (0.13, 0.98)	0.54 (0.27, 1.08)
	RERI	0.08 (-0.39, 0.47)	0.00 (-0.58, 0.52)	0.13 (-0.49, 0.72)	0.02 (-0.75, 0.59)

[a]Full population models adjusted for maternal age, race, education, mean dew point and having at least one day of pregnancy in spring or summer season

[b]Subpopulation models adjusted for maternal age, race, education and mean dew point.
